# Coenzyme Q_10_ Sunscreen Prevents Progression of Ultraviolet-Induced Skin Damage in Mice

**DOI:** 10.1155/2020/9039843

**Published:** 2020-08-19

**Authors:** Haiyou Wu, Zhangfeng Zhong, Sien Lin, Chuqun Qiu, Peitao Xie, Simin Lv, Liao Cui, Tie Wu

**Affiliations:** ^1^Department of Pharmacology, Guangdong Key Laboratory for Research and Development of Natural Drugs, Guangdong Medical University, Zhanjiang, Guangdong, China; ^2^Dongguan Institute for Food and Drug Control, Dongguan, Guangdong, China; ^3^Department of Orthopaedics and Traumatology and Li Ka Shing Institute of Health Sciences, Faculty of Medicine, The Chinese University of Hong Kong, Shatin, Hong Kong, China; ^4^The Joint Center of Guangdong Medical University and Guangdong Runhe Biotechnology Company for Co-Enzyme Q_10_ Research, Dongguan, Guangdong, China

## Abstract

The level of sun ultraviolet ray reaching the surface of the earth is increasing severely due to the rapid development of the society and environmental destruction. Excessive exposure to ultraviolet radiation causes skin damage and photoaging. Therefore, it is emerged to develop effective sunscreen to prevent ultraviolet-induced skin damage. This study was aimed at investigating the effects of Coenzyme Q_10_ (CoQ_10_) sunscreen on the prevention of ultraviolet B radiation- (UVB-) induced mouse skin damage. Three-month-old female mice were used, and they were randomly divided into four groups: control, model, CoQ_10_, and titanium dioxide (TiO_2_; positive control) groups. Our results showed that body weight, superoxide dismutase (SOD) and glutathione peroxidase (GSH-Px) activities, and DNA (cytosine-5)-methyltransferase 1 (DNMT1) protein expression were significantly decreased, while malondialdehyde (MDA) activity and metalloproteinase-1 (MMP-1) level were increased in UVB-treated mice. Besides, the stratum corneum was shed from the skin surface in the model group compared with the control group. In contrast, CoQ10 sunscreen prevented from UVB-induced skin damage, as well as reversing SOD, GSH-Px, and MDA activities, and MMP-1 and DNMT1 levels. Taken together, the current study provided further evidence on the prevention of UVB-induced skin damage by CoQ_10_ and its underlying mechanisms.

## 1. Introduction

The skin is the largest barrier that protects against the damage from the environmental risk factors and eventually results in skin aging. Skin aging can be categorized into intrinsic and extrinsic responses. The intrinsic skin aging occurs naturally as time passes [1], while extrinsic factors in skin aging are related to infection, water loss, and ultraviolet ray [2]. Even though only 5% of ultraviolet B radiation (UVB) light can reach the upper dermis of the skin, it is a key risk factor for extrinsic skin aging that affects dermal fibroblasts and skin microenvironment [3]. Collagen, a major component of extracellular matrix (ECM), is associated with extrinsic skin aging. Many studies have reported that collagen is degraded by matrix metalloproteases (MMPs), including MMP-1, MMP-8, and MMP-13 [4, 5]. In particular, MMP-1 is the predominant collagenase in the skin. Since wrinkle formation is evidenced by collagen degradation, the attenuation of MMP-1 activity is an important method for preventing skin aging [6, 7]. On the other hand, skin aging is also associated with decreased activity of antioxidant enzymes [8]. The system of oxidant and antioxidant tends to be balanced under normal conditions [9]. However, the levels of reactive oxygen species (ROS) are produced excessively when the skin is exposed to ultraviolet ray [10, 11]. Therefore, the scavenging capacity of the free radicals and the activities of antioxidant enzymes, superoxide dismutase (SOD) and glutathione peroxidase (GSH-Px), are reduced, while the amounts of free radicals, malondialdehyde (MDA), are increased.

Coenzyme Q_10_ (CoQ_10_, also known as ubiquinone) was found by Crane et al. in 1957 to be in the mitochondria of the beef heart, and broadly distributed in mammalian tissues [12, 13]. CoQ_10_, a necessary factor for healthy body, plays an important role in cardiovascular disorders and aging, including heart failure, hypertension, and endothelial dysfunction [14]. Growing evidence showed that CoQ_10_ also has a potential role for the prevention and treatment of heart ailments by improving cellular bioenergetics via scavenging free radicals [15]. With regard to ultraviolet A radiation- (UVA-) induced skin aging, CoQ_10_ might be a useful preventive medication against skin photoaging [16]. Among CoQ_10_-loaded conventional carriers, ultrasmall lipid nanoparticles containing CoQ_10_ exhibited reduced capacity in free radical formation compared with non-nanocarrier-treated cells. Therefore, ultrasmall lipid nanoparticles containing CoQ_10_ were shown to be suitable to increase the antioxidant capacity of the skin [17]. Moreover, CoQ_10_ significantly reduced the levels of myeloperoxidase (MPO), phospholipase A2 (PLA2), and MDA, while it increased SOD levels *in vivo* and *in vitro* [18]. A study found that CoQ_10_ might rejuvenate a wrinkled skin through inhibiting the degradation of dermal fiber components and stimulating the paracrine of dermal fiber via upregulation of interleukin- (IL-) 6 and MMP secretion [19]. As the systemic delivery of antioxidants to the skin is poor, it may be beneficial to penetrate the skin with sufficient amount of topical application [20, 21]. The present study explored the preventive effects of CoQ_10_ sunscreen against skin damage induced by UVB as topical application on a mouse skin.

## 2. Materials and Methods

### 2.1. Materials

CoQ_10_ was provided by Runhe Biology Co. (Guangzhou, China), while titanium dioxide (TiO_2_) was purchased from Kemao Chemical Co. (Dongguan, China). Ointment base was made by our laboratory, which contained purified water, petrolatum, polysorbate 80, and cetostearyl alcohol and did not contain any drug.

### 2.2. Animals and Treatments

This study was carried out according to the Guide for the Care and Use of Laboratory Animals of Guangdong Laboratory Animal Monitoring Institute, the National Laboratory Animal Monitoring Institute of China. All the procedures performed were in accordance with the ethical standards of the Academic Committee on the Ethics of Animal Experiments of Guangdong Medical University. 36 specific pathogen-free female Kunming mice were acclimatized to local vivarium conditions (temperature 24-26°C, humidity 67%) and allowed to free access of water and diets containing 1.11% calcium and 0.74% phosphorus. The average weight of mice was about 27.66 g ± 0.56.

CoQ_10_ sunscreen was composed of CoQ_10_ and ointment base at the concentration of 10 mg/g. TiO_2_ sunscreen was used as a positive control and composed of TiO_2_ and ointment base at the concentration of 50 mg/g [18, 19]. 36 mice were randomly divided into four groups: control group (*n* = 9), aging model group (ointment base without additives; *n* = 9), CoQ_10_ group (CoQ_10_ sunscreen; *n* = 9), and TiO_2_ group (TiO_2_ sunscreen; *n* = 9). The hair on the back of each mouse was shaved, and 0.5 g of ointment was topically applied to 3 × 3 cm^2^ of the skin once daily for 8 weeks. Except the control group, the other groups were exposed to ultraviolet B radiation (UVB; 303 nm and 1522.7 *μ*W/cm^2^) under diffused UV light (Sentry Optronics CORP, Taiwan) for 30 mins every day.

### 2.3. Sample Collection

All the mice were weighed weekly. At the end of the treatment, the mice were sacrificed, and blood was collected from the eyeballs. Firstly, the serum was collected from the blood by centrifugation, and it was used for biochemical assays. And then, the dorsal skin, heart, liver, kidney, and brain were dissected, weighed, and normalized by body weight. Finally, dorsal skin tissues were also collected for histological, biochemical, and quantitative real-time PCR analyses.

### 2.4. Biochemical Analysis

The degree of skin damage exposed to UVB could be determined by evaluating the activities of MDA, SOD, and GSH-Px [22]. They are the most frequently used biomarkers of oxidative stress (imbalance between oxidant and antioxidant systems) in the skin tissues. MDA levels in the dorsal skin were measured using a MDA detection kit (Nanjing Jiancheng Bioengineering Institute, Nanjing, China) according to the instructions of the manufacturer. The activities of SOD and GSH-Px in the skin tissues were detected using a commercial kit (Nanjing Jiancheng Bioengineering Institute, Nanjing, China).

### 2.5. Histological Analysis

Each part of the skin samples (1 × 1 cm^2^) was fixed with 4% paraformaldehyde for Van Gieson (VG) and hematoxylin and eosin (H&E) staining. H&E staining was used to assess the skin structure alteration, while VG staining was applied to detect the presence of collagen fibers. All the stained skin specimens were observed and photographed using an optical microscope (Nikon Eclipse Boi, Nikon Corporation, Japan).

### 2.6. Quantitative Real-Time PCR

Total RNA was extracted from the dorsal skin tissues using Trizol reagent (TaKaRa Bio, Otsu, Japan) as recommended by the manufacturer. Total RNA was reverse transcribed to cDNA using a commercial kit (Takara Bio, Otsu, Japan) according to the protocol of the manufacturer. Target genes were amplified with SYBR Premix Ex Taq™ (Takara Bio, Otsu, Japan) using a PikoReal 96 Real-Time PCR System (Thermo Fisher Scientific, Vantaa, Finland). The sequences of the forward and reverse primers (Shenggong Bio. Co., Shanghai, China) are shown in Table 1. To confirm the specificity of the amplification, PCR products were evaluated by melting curve analysis. mRNA expression was determined based on the cycle threshold values, which was normalized to that of *β*-actin, and calculated using the 2^−*ΔΔ*CT^ method [23].

### 2.7. Western Blotting

The total protein was extracted from the dorsal skin tissues with ice-cold lysis buffer. The protein concentrations of the lysates were measured by the bicinchoninic acid kit (Pierce, France). An equal amount of proteins was used and separated by SDS-PAGE gels and then transferred onto the nitrocellulose membranes. Then, the membranes were incubated with primary DNMT1 antibody (Cell Signaling, USA), and anti-rabbit-HRP antibody (Cell Signaling, USA). The blots were developed by enhanced chemiluminescence (GE Healthcare Life Sciences, USA) with a ChemiDoc™ MP System (Bio-Rad Laboratories, USA). *α*-Tubulin antibody (Cell Signaling, USA) was used as a housekeeping control.

### 2.8. Statistical Analysis

The results were expressed as the mean ± standard deviation (SD). The data were analyzed using SPSS 17.0 software (SPSS Inc., Chicago, IL, USA). The significance of differences between groups was evaluated by one-way or two-way ANOVA, and *p* < 0.05 was considered statistically significant.

## 3. Results

### 3.1. CoQ_10_ Sunscreen Slightly Altered Body and Organ Weights in UVB-Treated Mice

**Figure 1:**
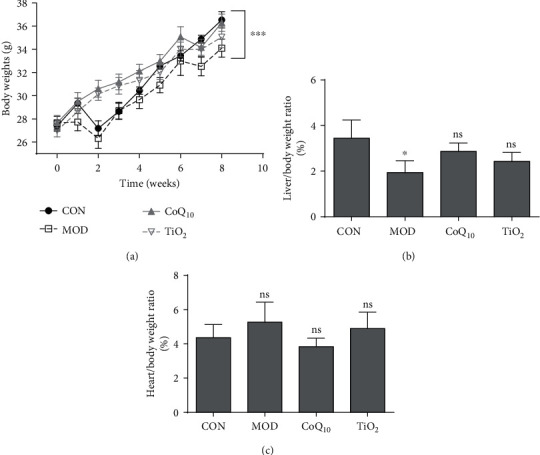
Body and organ weight changes with Coenzyme Q_10_ (CoQ_10_) sunscreen treatment in response to ultraviolet B radiation (UVB). The weights of the (a) body, (b) liver, and (c) heart in response to UVB. CON: control group without exposing to UVB; MOD: model group with ointment base exposed to UVB; CoQ_10_: treatment with CoQ_10_ sunscreen exposed to UVB; TiO_2_: positive control with titanium dioxide (TiO_2_) sunscreen exposed to UVB. *n* ≥ 3 for each group. Results were shown as the mean ± SD. ^∗^*p* < 0.05 vs. CON, ^∗∗∗^*p* < 0.001 vs. MOD.

As shown in Figure 1(a), the body weights of the model mice were significantly decreased compared to those of the control mice. In contrast, the body weights of CoQ_10_ and TiO_2_ mice were significantly increased compared to those of the model mice. The results also showed that liver weight was significantly decreased when the mice were exposed to UVB compared to control mice (Figure 1(b)). However, there were no statistical differences in other groups. The heart weight was also not affected by CoQ_10_ and TiO_2_ sunscreen compared with control mice (Figure 1(c)).

### 3.2. CoQ_10_ Sunscreen Altered Antioxidant Enzyme Activities in UVB-Treated Skin

**Figure 2:**
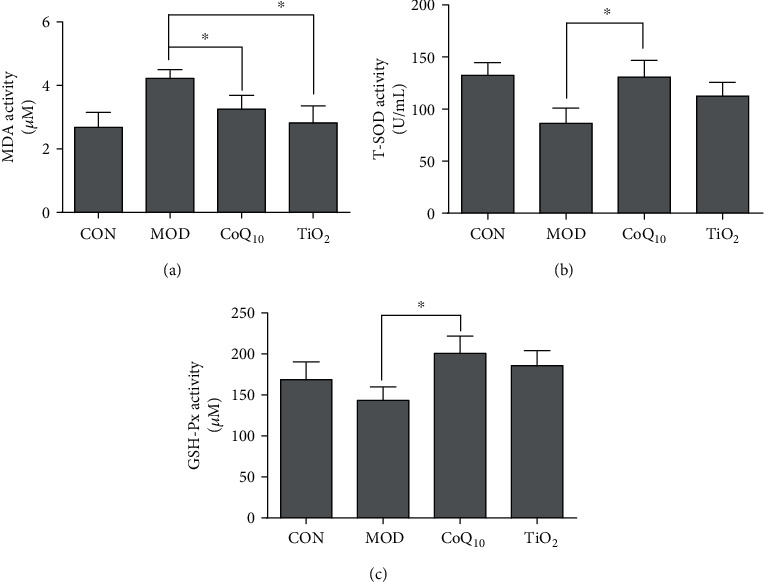
The antioxidant enzyme activities were altered by Coenzyme Q_10_ (CoQ_10_) sunscreen treatment in ultraviolet B radiation- (UVB-) treated skin. The activities of (a) malondialdehyde (MDA), (b) superoxide dismutase (SOD), and (c) glutathione peroxidase (GSH-Px) in the UVB-treated skin tissue in response to CoQ_10_ sunscreen treatment. CON: control group without exposing to UVB; MOD: model group with ointment base exposed to UVB; CoQ_10_: treatment with CoQ_10_ sunscreen exposed to UVB; TiO_2_: positive control with titanium dioxide (TiO_2_) sunscreen exposed to UVB. *n* ≥ 3 for each group. Results were shown as the mean ± SD. ^∗^*p* < 0.05 vs. MOD.

We investigated the activities of antioxidant enzymes in UVB-treated skin in response to CoQ_10_ sunscreen treatment. Compared with control group, the MDA activity was increased in the dorsal skin of the model group (Figure 2(a)). Interestingly, the MDA activity was significantly decreased in the CoQ_10_ and TiO_2_ groups compared with the model group. Moreover, the activities of T-SOD and GSH-Px were decreased in the dorsal skin of the model group compared with the control group (Figures 2(b) and 2(c)). The reductions of these activities were significantly attenuated in the CoQ_10_ group compared to the model group. Similarly, the TiO_2_ group, as a positive control, also slightly attenuated these reductions compared to the model group (Figures 2(b) and 2(c)).

### 3.3. CoQ_10_ Sunscreen Protected the Epidermis in UVB-Treated Skin

**Figure 3:**
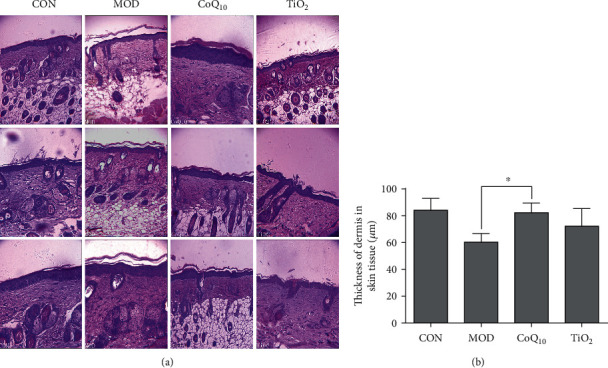
Coenzyme Q_10_ (CoQ_10_) restored ultraviolet B radiation- (UVB-) induced damage in the epidermis of the skin. (a) Hematoxylin and eosin (H&E) staining of the epidermis and dermis on the mouse skin. The picture was captured at 10x magnification using an electron microscope. (b) The thickness of the dermis was measured in response to CoQ_10_ treatment. CON: control group without exposing to UVB; MOD: model group with ointment base exposed to UVB; CoQ_10_: treatment with CoQ_10_ sunscreen exposed to UVB; TiO_2_: positive control with titanium dioxide (TiO_2_) sunscreen exposed to UVB. *n* ≥ 3. Results were shown as the mean ± SD. ^∗^*p* < 0.05 vs. MOD.

To investigate the effect of CoQ_10_ sunscreen on the epidermis in the UVB-treated skin, H&E staining was used, and the thickness of the dermis was also assessed. Our results showed that the epidermis of control mice was unbroken and its stratum corneum was not shed from the skin surface, but the epidermis of model mice was injured and its stratum corneum was shed from the skin surface obviously (Figure 3(a)). Compared with the model group, there were no differences in the CoQ_10_ and TiO_2_ groups. Interestingly, the skin of CoQ_10_ mice was not injured and its stratum corneum was not shed from the skin surface, but the skin of TiO_2_ mice appeared to be a little bit broken. Furthermore, the stratum corneum of TiO_2_ mice was shed from the skin surface slightly, but it looked healthier than that of model mice. In addition, the thickness of the dermis in the model mice was decreased compared with control mice, and this reduction in thickness was significantly prevented in CoQ_10_ mice (Figure 3(b)).

### 3.4. CoQ10 Sunscreen Prevented the Degradation of Collagen in UVB-Treated Skin

**Figure 4:**
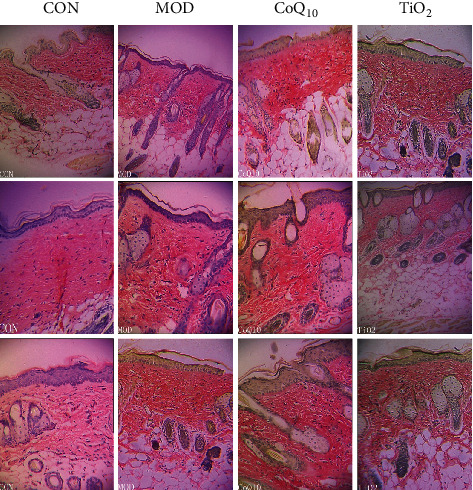
Coenzyme Q_10_ (CoQ_10_) sunscreen restored collagen degradation in ultraviolet B radiation- (UVB-) treated skin. Van Gieson staining was used to detect collagen on the skin. The picture was captured at 10x magnification using an electron microscope. CON: control group without exposing to UVB; MOD; model group with ointment base exposed to UVB; CoQ_10_: treatment with CoQ_10_ sunscreen exposed to UVB; TiO_2_: positive control with titanium dioxide (TiO_2_) sunscreen exposed to UVB.

In order to investigate the effect of CoQ_10_ sunscreen on collagen degradation in the UVB-treated skin, Van Gieson staining was performed. As shown in Figure 4, we found that the collagen fibers of control mice were deposited neatly, while there were less collagen fibers in the model mice that were rowed irregularly. Moreover, its stratum corneum was shed from the skin surface obviously in the model group. Compared with the model group, the skin of CoQ_10_ mice was not injured and its collagen fibers were rowed regularly. However, the skin of TiO_2_ mice appeared to be a little bit broken, its stratum corneum was shed from the skin surface slightly, and its collagen fibers were rowed regularly.

### 3.5. CoQ_10_ Sunscreen Modulated MMP-1 Expression in UVB-Treated Skin

**Figure 5:**
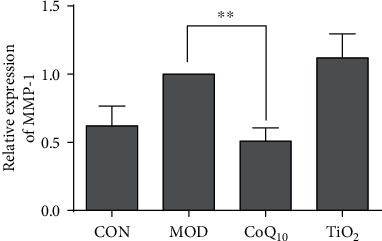
Coenzyme Q_10_ (CoQ_10_) sunscreen altered MMP-1 mRNA level in ultraviolet B radiation- (UVB-) treated skin. The MMP-1 level of the mouse skin was measured by real-time PCR. CON: control group without exposing to UVB; MOD: model group with ointment base exposed to UVB; CoQ_10_: treatment with CoQ_10_ sunscreen exposed to UVB; TiO_2_: positive control with titanium dioxide (TiO_2_) sunscreen exposed to UVB. *n* ≥ 3. Results were shown as the mean ± SD. ^∗∗^*p* < 0.01 vs. MOD.

Skin collagen degradation is mainly regulated by MMP-1 [24]. We found that the MMP-1 mRNA level of the model group was increased compared with the control group (Figure 5). CoQ_10_ treatment significantly attenuated this upregulation of MMP-1 level induced by UVB. Conversely, the MMP-1 level was not decreased in the TiO_2_ group compared with the model group.

### 3.6. CoQ_10_ Sunscreen Prevented DNMT1 Downregulation in UVB-Treated Skin

**Figure 6:**
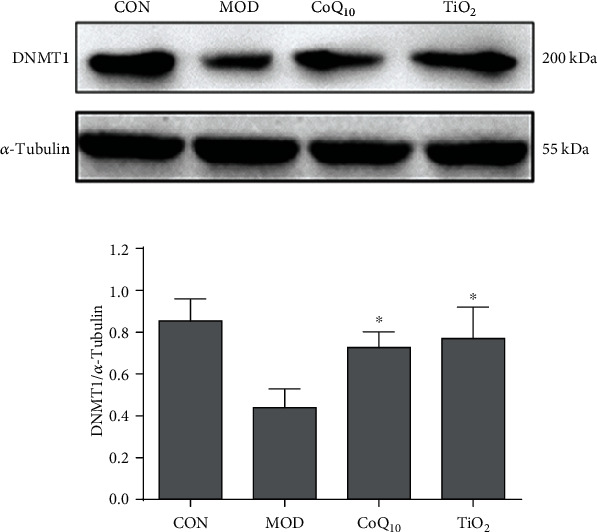
Coenzyme Q_10_ (CoQ_10_) sunscreen altered DNMT1 protein expression in ultraviolet B radiation- (UVB-) treated skin. Immunoblots and representative graph showing the expression of DNMT1. The expression of DNMT1 was measured by western blotting. CON: control group without exposing to UVB; MOD: model group with ointment base exposed to UVB; CoQ_10_: treatment with CoQ_10_ sunscreen exposed to UVB; TiO_2_: positive control with titanium dioxide (TiO_2_) sunscreen exposed to UVB. *n* ≥ 3. Results were shown as the mean ± SD. ^∗^*p* < 0.05 vs. MOD.

DNMT1 was shown to be associated with UV-induced photoaging [25]. Next, the DNMT1 protein expression was also investigated in the UVB-treated skin. Our results showed that DNMT1 expression was decreased in the model group compared to the control group (Figure 6). Besides, CoQ_10_ and TiO_2_ sunscreen treatment significantly suppressed this downregulation induced by UVB.

## 4. Discussion

The skin is regarded as the first line of defense against infection and environmental factors, such as ultraviolet (UV) radiation. Sunlight is the main source of UV radiation, which can induce skin senescence, inflammation, aging, and cancer [26]. Therefore, protecting the skin with sunscreen is very important to avoid skin damage. CoQ_10_ was shown to be an antioxidant molecule that could prevent UV-induced DNA damage [19]. In this study, we further investigated the preventive effects of CoQ_10_ sunscreen on UVB-induced skin damage in mice and its underlying mechanisms.

In this study, we found that the mouse skin was damaged by UVB. This was shown by the decrease in the growth rate of body and liver weights in UVB-treated mice. Besides, UVB decreased the activities of SOD and GSH-Px and increased MDA activity in the mouse skin. This suggested that the balance between oxidant and antioxidant systems was impaired when the mouse skin was exposed to UVB only. During the aging process, the skin dermis becomes thin and damaged [27], due to the degradation of the collagen matrix [28, 29]. We also showed that the stratum corneum of the mouse skin that was exposed to UVB was shed from the skin surface through regulating collagen via upregulation of MMP-1 expression. Taken together, our mouse UVB model could be a suitable model for skin aging.

In the current study, we demonstrated that topical application of CoQ_10_ sunscreen could alleviate the alterations of collagen in the mouse skin induced by UVB, and the stratum corneum of the mouse skin was not shed from the skin surface. MMP-1 expression was shown to be increased with age, which is a major factor that causes collagen breakdown and wrinkling problems [30, 31]. Indeed, aging is the primary consequence of aerobic metabolism, which produces excess ROS and exceeds the capacity of cellular antioxidant defense [32]. Therefore, oxidants are important mediators of aging [33]. In fact, ROS production is also related to age-associated upregulation of MMP-1 [27]. Interestingly, we showed that CoQ_10_ sunscreen treatment could inhibit MMP-1 upregulation and collagen degradation induced by UVB in the mouse skin. Similarly, CoQ_10_ sunscreen was also shown to reduce MMP-1 levels in dermal fibroblasts [19]. Moreover, antioxidant enzymes in the skin, including SOD and GSH-Px, can counteract ROS [34]. Our results showed that the activities of SOD and GSH-Px were significantly increased by CoQ_10_ sunscreen in the mouse skin. Furthermore, MDA is a biomarker of cell membrane damage caused by free radicals [35]. We found that CoQ_10_ sunscreen could reduce MDA activity induced by UVB. Taken together, we suggest that CoQ_10_ sunscreen has antioxidant activities against UVB damage in the mouse skin and prevents from collagen degradation by suppressing MDA activity and MMP-1 levels and enhancing SOD and GSH-Px activities.

## 5. Conclusions

In conclusion, our findings indicated that topical application of CoQ_10_ sunscreen prevents UVB-induced skin damage by enhancing the antioxidant capacity of the skin and delays the breakdown of collagen through suppressing MMP-1 level and MDA activity in the mouse skin. Therefore, we suggest that CoQ_10_ sunscreen might have beneficial effects in antiaging, and topical application of CoQ_10_ sunscreen could be potential to protect against UVB-induced photoaging.

## Figures and Tables

**Table 1:** Primer sequences for quantitative real-time-polymerase chain reaction.

Gene	Forward primer sequences (5′ to 3′)	Reverse primer sequences (5′ to 3′)
*β-Actin*	GCCAACCGTGAAAAGATGAC	ACCAGAGGCATACAGGGACAG
*MMP-1*	CCCAAATCCCATCCAGCCAA	ATTAAATTGAGCTCAGGTTCTGGC

## Data Availability

The experimental data used to support the findings of this study are available from the corresponding author upon request.

## Abbreviations

CoQ_10_: Coenzyme Q_10_
DNMT1: DNA (cytosine-5)-methyltransferase 1
ECM: Extracellular matrix
GSH-Px: Glutathione peroxidase
H&E: Hematoxylin and eosin
IL: Interleukin
MDA: Malondialdehyde
MMP-1: Metalloproteinase-1
MMPs: Matrix metalloproteases
MPO: Myeloperoxidase
PLA2: Phospholipase A2
ROS: Reactive oxygen species
SD: Standard deviation
SOD: Superoxide dismutase
TiO_2_: Titanium dioxide
UV: Ultraviolet
UVA: Ultraviolet A radiation
UVB: Ultraviolet B radiation
VG: Van Gieson.

## References

[1] Yaar M., Eller M. S., Gilchrest B. A. (2002). Fifty years of skin aging. *Journal of Investigative Dermatology Symposium Proceedings*.

[2] Chung J. H. (2003). Photoaging in Asians. *Photodermatology, Photoimmunology and Photomedicine*.

[3] BRULS W. I. E. L. A. G., WEELDEN H. U. I. B., LEUN J. A. N. C. (1984). Transmission of UV-radiation through human epidermal layers as a factor influencing the minimal erythema dose. *Photochemistry and Photobiology*.

[4] Varani J., Dame M. K., Rittie L. (2006). Decreased Collagen Production in Chronologically Aged Skin: Roles of Age- Dependent Alteration in Fibroblast Function and Defective Mechanical Stimulation. *The American Journal of Pathology*.

[5] Pilcher B. K., Sudbeck B. D., Dumin J. A., Welgus H. G., Parks W. C. (1998). Collagenase-1 and collagen in epidermal repair. *Archives of Dermatological Research*.

[6] Xia W., Quan T., Hammerberg C., Voorhees J. J., Fisher G. J. (2015). A mouse model of skin aging: fragmentation of dermal collagen fibrils and reduced fibroblast spreading due to expression of human matrix metalloproteinase-1. *Journal of Dermatological Science*.

[7] Fligiel S. E. G., Varani J., Datta S. C., Kang S., Fisher G. J., Voorhees J. J. (2003). Collagen Degradation in Aged/Photodamaged Skin In Vivo and After Exposure to Matrix Metalloproteinase-1 In Vitro. *Journal of Investigative Dermatology*.

[8] Gutteridge J. M. C., Halliwell B. (2006). Free radicals and antioxidants in the year 2000. A historical look to the future. *Annals of the New York Academy of Sciences*.

[9] Thiele J. J., Schroeter C., Hsieh S. N., Podda M., Packer L. (2001). The antioxidant network of the stratum corneum. *Current Problems in Dermatology*.

[10] Guyton K. Z., Gorospe M., Wang X. (1998). Age-related changes in activation of mitogen-activated protein kinase cascades by oxidative stress. *Journal of Investigative Dermatology Symposium Proceedings*.

[11] Harman D. (2006). Free radical theory of aging: an update: increasing the functional life span. *Annals of the New York Academy of Sciences*.

[12] Prakash S., Sunitha J., Hans M. (2010). Role of coenzyme Q(10) as an antioxidant and bioenergizer in periodontal diseases. *Indian Journal of Pharmacology*.

[13] Potgieter M., Pretorius E., Pepper M. S. (2013). Primary and secondary coenzyme Q10 deficiency: the role of therapeutic supplementation. *Nutrition Reviews*.

[14] Yang Y.-K., Wang L.-P., Chen L. (2015). Coenzyme Q10 treatment of cardiovascular disorders of ageing including heart failure, hypertension and endothelial dysfunction. *Clinica Chimica Acta*.

[15] Kumar A., Kaur H., Devi P., Mohan V. (2009). Role of coenzyme Q10 (CoQ10) in cardiac disease, hypertension and Meniere-like syndrome. *Pharmacology & Therapeutics*.

[16] Tanino Y., Budiyanto A., Ueda M. (2005). Decrease of antioxidants and the formation of oxidized diacylglycerol in mouse skin caused by UV irradiation. *Journal of Dermatological Science Supplement*.

[17] Lohan S. B., Bauersachs S., Ahlberg S. (2015). Ultra-small lipid nanoparticles promote the penetration of coenzyme Q10 in skin cells and counteract oxidative stress. *European Journal of Pharmaceutics and Biopharmaceutics*.

[18] Choi B. S., Song H. S., Kim H. R. (2009). Effect of coenzyme Q10 on cutaneous healing in skin-incised mice. *Archives of Pharmacal Research*.

[19] Inui M., Ooe M., Fujii K., Matsunaka H., Yoshida M., Ichihashi M. (2008). Mechanisms of inhibitory effects of CoQ10 on UVB-induced wrinkle formation in vitro and in vivo. *Biofactors*.

[20] Werninghaus K., Meydani M., Bhawan J., Margolis R., Blumberg J. B., Gilchrest B. A. (1994). Evaluation of the photoprotective effect of oral vitamin E supplementation. *Archives of Dermatology*.

[21] Ahn S. M., Hwang J. S., Lee S. H. (2007). Fructose 1,6-diphosphate alleviates UV-induced oxidative skin damage in hairless mice. *Biological & Pharmaceutical Bulletin*.

[22] Hamdan D., El-Readi M. Z., Tahrani A. (2011). Chemical composition and biological activity of Citrus jambhiri Lush. *Food Chemistry*.

[23] Schmittgen T. D., Zakrajsek B. A., Mills A. G., Gorn V., Singer M. J., Reed M. W. (2000). Quantitative reverse transcription-polymerase chain reaction to study mRNA decay: comparison of endpoint and real-time methods. *Analytical Biochemistry*.

[24] Van Doren S. R. (2015). Matrix metalloproteinase interactions with collagen and elastin. *Matrix Biology*.

[25] Yi Y., Xie H., Xiao X. (2018). Ultraviolet A irradiation induces senescence in human dermal fibroblasts by down-regulating DNMT1 via ZEB1. *Aging*.

[26] Cleaver J. E., Crowley E. (2002). UV damage, DNA repair and skin carcinogenesis. *Frontiers in Bioscience*.

[27] Fisher G. J., Varani J., Voorhees J. J. (2008). Looking older: fibroblast collapse and therapeutic implications. *Archives of Dermatology*.

[28] Fossel M. (2002). Cell senescence in human aging and disease. *Annals of the New York Academy of Sciences*.

[29] Bonta M., Daina L., Mutiu G. (2013). The process of ageing reflected by histological changes in the skin. *Romanian Journal of Morphology and Embryology*.

[30] Mawal-Dewan M., Lorenzini A., Frisoni L., Zhang H., Cristofalo V. J., Sell C. (2002). Regulation of collagenase expression during replicative senescence in human fibroblasts by Akt-forkhead signaling. *Journal of Biological Chemistry*.

[31] Kumar S., Vinci J. M., Millis A. J. T., Baglioni C. (1993). Expression of interleukin-1*α* and *β* in early passage fibroblasts from aging individuals. *Experimental Gerontology*.

[32] Harman D. (1992). Free radical theory of aging. *Mutation Research/DNAging*.

[33] Cutler R. G. (1991). Antioxidants and aging. *The American Journal of Clinical Nutrition*.

[34] Truong V.-L., Bak M.-J., Jun M., Kong A.-N. T., Ho C.-T., Jeong W.-S. (2014). Antioxidant defense and hepatoprotection by procyanidins from almond (Prunus amygdalus) skins. *Journal of Agricultural and Food Chemistry*.

[35] Bharathy N., Kumr C. N., Kumar A. A. (2015). Antioxidant status in neonatal jaundice before and after phototherapy. *Journal of Pharmacy and Bioallied Sciences*.

